# Towards Precision Medicine in Obesity: Genetic Copy Number Variations Profiling Linked to Specific Metabolic Dysregulation Patterns

**DOI:** 10.3390/ijms26104782

**Published:** 2025-05-16

**Authors:** Ivona Mitu, Iuliu Ivanov, Loredana Dragoș, Elena Nisioi, Daniela-Cristina Dimitriu, Larisa-Ionela Miftode, Otilia Frăsinariu, Laura-Mihaela Trandafir, Roxana Popescu, Daniela Jitaru

**Affiliations:** 1 Department of Morpho-Functional Sciences II, University of Medicine and Pharmacy “Grigore T. Popa”, 700115 Iasi, Romania; ivona.mitu@umfiasi.ro; 2 Molecular Diagnosis Department, Regional Institute of Oncology, 700483 Iasi, Romania; iuliuic@gmail.com (I.I.); nisioielena@gmail.com (E.N.); danielajitaru@yahoo.com (D.J.); 3 Department of Infectious Diseases, University of Medicine and Pharmacy “Grigore T. Popa”, 700115 Iasi, Romania; ionela-larisa.miftode@umfiasi.ro; 4 Department of Mother and Child, University of Medicine and Pharmacy “Grigore T. Popa”, 700115 Iasi, Romania; frasinariu.otilia@umfiasi.ro (O.F.); laura.trandafir@umfiasi.ro (L.-M.T.); 5 Department of Medical Genetics, University of Medicine and Pharmacy “Grigore T. Popa”, 700115 Iasi, Romania; roxana.popescu2014@gmail.com

**Keywords:** obesity, genetic panel, MLPA, copy number variations, metabolic phenotype

## Abstract

This study aimed to identify and analyse the copy number variations (CNVs) in the genes involved in the pathophysiology of obesity and correlate these findings with the phenotypic manifestations. Genetic screening of 59 apparently healthy individuals with elevated adipose tissue percentages was performed, assessing the duplications and deletions of obesity-related genes through the MLPA (Multiplex Ligation-dependent Probe Amplification) technique. Clinical and metabolic parameters, including insulin, HOMA-IR, leptin, and adiponectin levels, were measured to better describe the obesity profiles of the participants in this study. In our research, 11.86% of the subjects presented with genetic alterations in obesity-associated genes, with 16% of these modifications involving concurrent duplications in *SEZ6L2-1* and *SH2B1-2*, linked to doubled insulin and tripled HOMA-IR levels. However, the same duplications were associated with a reduced trunk adipose tissue percentage (but not BMI), suggesting leptin signalling modulation. Duplications were more frequent in the metabolically unhealthy obese patients, resulting in a higher relative risk of an obese metabolically unhealthy diagnosis (1.85-fold increased risk in subjects with *SEZ6L2-1*/*SH2B1-2* duplications, *p* = 0.52). No duplications or deletions were reported in the non-obese patient groups, defined according to the BMI criteria. A partial *LEPR* deletion was identified in one patient, associated with severe insulin resistance (second-highest HOMA-IR in the cohort). Another subject presented with 11 duplications (7 in LEPR) and reported the lowest adiponectin and second-highest leptin levels among the genetically altered subjects. The genetic profiles revealed complex associations between the CNVs and obesity phenotypes, highlighting the potential for early risk stratification. Despite the interpretative challenges, identifying the genetic predispositions could significantly predict cardiometabolic risk and be used to map personalised interventions to possibly modulate phenotypic expression.

## 1. Introduction

Copy number variations (CNVs) primarily refer to deletions or duplications of specific DNA segments and represent an important component of human genetic diversity. Significant progress has been made in understanding the role of genetic alterations in the aetiology of obesity, with numerous genes have been identified as being involved in the complex pathophysiological processes of this condition. CNVs can modulate gene expression through multiple indirect mechanisms, such as the unmarking of recessive alleles, position effects altering the genomic architecture, and the disruption of regulatory elements affecting allelic communication [1]. Deletions and duplications may be either normal or pathogenic; the challenge remains in estimating whether it they are benign or could compromise fundamental cellular mechanisms, resulting in disease. While genetic duplications have a milder phenotypic effect compared to deletions, both variants may modulate both polygenic traits and sporadic phenotypic expressions.

From a genetic standpoint, obesity is classified into two main categories: monogenic and polygenic. Monogenic obesity is rare, severe, manifests early in life, and involves chromosomal deletions or single-gene defects. In contrast, common obesity is polygenic in nature, considered to result from hundreds of polymorphisms, each contributing a small individual effect. To date, over 500 genes have been associated with obesity, among which eight are considered particularly relevant to obesity’s pathophysiology. These include the genes encoding for leptin (LEP), leptin receptor (LEPR), proopiomelanocortin (POMC), proprotein convertase subtilisin/kexin type 1 (PCSK1), melanocortin-4 receptor (MC4R), single-minded homolog 1 (SIM1), brain-derived neurotrophic factor (BDNF), and neurotrophic tyrosine kinase receptor type 2 (NTRK2) [2]. Recent genome-wide studies have identified obesity-associated CNVs at loci, such as 16p12.3, 11q11, and 10q26.3, which may disrupt energy homeostasis and adipogenesis pathways, offering potential diagnostic and therapeutic insights [3]. Beyond its structural genomic alterations, obesity is characterised by metabolic dysfunction and chronic inflammation, documented by elevated BMI and CRP levels and leukocyte counts, alongside epigenetic dysregulation. Also, global DNA hypomethylation and the overexpression of proinflammatory genes in obese patients suggest that CNV-driven genomic instability may interact with epigenetic mechanisms to amplify metabolic dysregulation. Together, these findings highlight the need to integrate CNVs profiling with epigenetic and inflammatory biomarkers (such as CRP and methylation status) to improve patient stratification and personalise obesity management strategies [4].

Approximately 60% of acquired obesity is polygenic. Current GWASs involve large patient cohorts (up to 800,000 participants), thereby providing substantially greater statistical power for identifying an increasing number of loci compared to initial studies that included only about 5000 patients. Therefore, the likelihood of detecting loci with even modest effects on obesity is significantly increased. To date, the reported data explain 6% of BMI variation [5], 0.58% of total body fat percentage variation [6], 0.23% of total lean mass percentage variation [7], and 0.1–4.4% of the variation associated with the differentiation and development of ectopic adipose tissue [8]. Notably, four loci that demonstrate significant effects on body fat percentage correspond to the genes involved in insulin receptor signalling (*IRS1* and *GRB14*) and the growth hormone/insulin-like growth factor-1 pathway (*IGF2BP1* and *PICK1*); both are related to insulin receptor signalling [6]. However, translating these GWAS-identified loci into novel biological insights remains a major scientific challenge.

Several techniques can be utilised to assess genetic alterations, among which Multiplex Ligation-dependent Probe Amplification (MLPA) stands out as a technique that enables the investigation of multiple loci in a single test and shows potential as an important screening tool for large patient cohorts. This study aimed to test patients with different clinical obesity phenotypes for duplications or deletions in key obesity-related genes and connect the results with the markers used to describe an obesity profile, like adiponectin, leptin, and insulin.

## 2. Results

**Figure 1:**
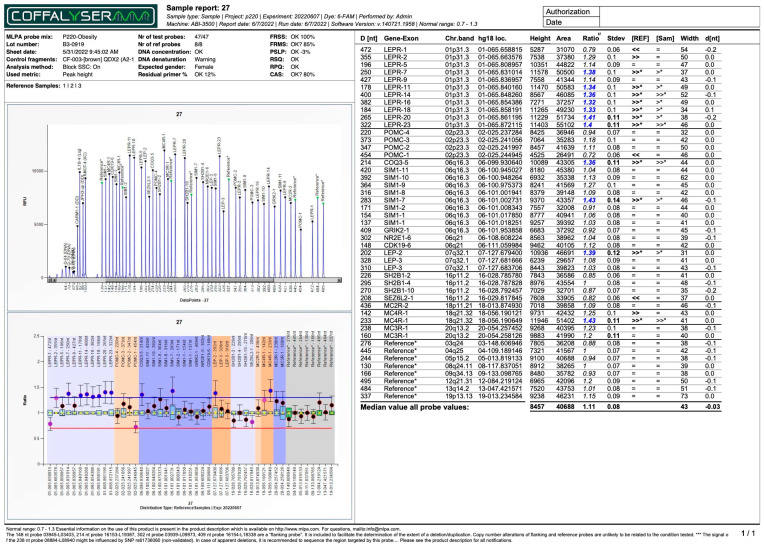
MLPA report for patient number 27. The “*” in bold denotes a Ratio value outside the normal range (0.7–1.3), indicating potential copy number variation.

Among the 59 subjects, 7 (11.86%) exhibited genetic alterations in the form of duplications or deletions within the exons of genes associated with the pathophysiology of obesity. Duplications, ranging in number from 1 to 11, were found in all seven patients, while deletions were found in only two patients. The patient with 11 duplications (Figure 1) exhibited most of them in the *LEPR* gene and had the highest percentage of adipose tissue, both in the trunk region and throughout their whole body. This patient also displayed the lowest adiponectin levels compared to the other six patients, while their leptin levels were among the highest. The anthropometric and biochemical data for this study’s cohort are presented in Table 1, while Table 2 provides detailed information regarding the patients with duplications and/or deletions, as well as the affected regions.

**Figure 2:**
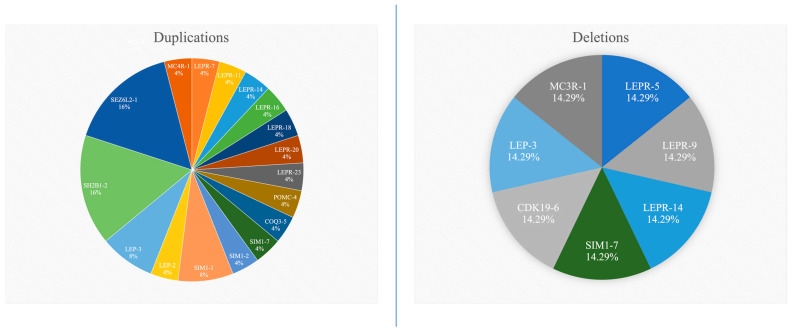
Percentage representation of duplications and deletions within this study’s population.

**Figure 3:**
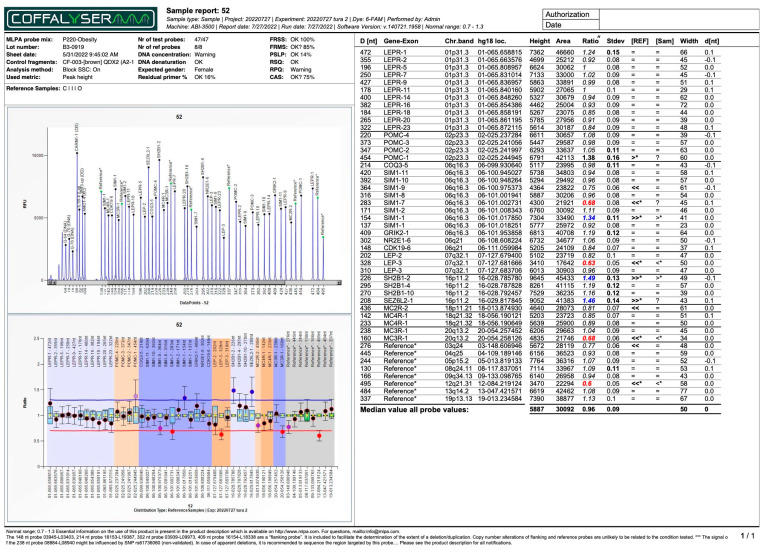
MLPA report for patient number 52. The “*” in bold denotes a Ratio value outside the normal range (0.7–1.3), indicating potential copy number variation.

A total of nine exons of the *LEPR* gene, two exons of the *LEP* gene, three exons of the *SIM1* gene, and one exon each from the *POMC*, *COQ3*, *CDK19*, *SH2B1*, *SEZ6L2*, *MC4R*, and *MC3R* genes exhibited alterations. No CNVs were detected in the examined exons of the *MC2R* (exon 2), *GRIK2* (exon 1), and *NR2E1* (exon 6) genes. Among the identified duplications, exon 1 of the *SEZ6L2* gene had the highest percentage (16%—four subjects), together with exon 2 of the *SH2B1* gene (16%—four subjects), followed by exon 3 of the *LEP* gene (8%—two subjects), and exon 1 of the *SIM1* gene (8%—two subjects). The genes, and exons of, *LEP-3*, *SIM1-7*, and *LEPR-14* exhibited both duplications and/or deletions in this study’s group, as shown in Figure 2. The most common duplication (*SH2B1-2*/*SEZ6L2-1*) can be found in Figure 3. All the other MLPA reports for the subjects that presented with modifications are provided in the Supplementary Materials (Figures S1–S5).

**Figure 4:**
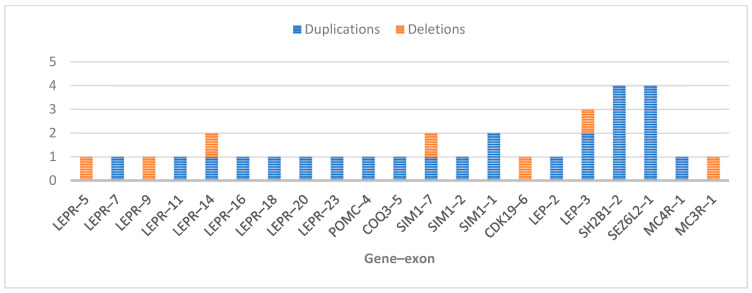
The count of duplications/deletions identified per analysed gene–exon in this study.

The number of duplications or deletions observed at the gene–exon level for each analysed gene is illustrated in Figure 4.

In the obese and metabolically unhealthy group, duplications and/or deletions were present in 5 out of 37 subjects (13.51%—patients no. 27, 43, 45, 47, and 52), while in the obese but metabolically healthy group, duplications and/or deletions were found in 2 out of 15 subjects (13.33%—patients no. 4 and 53). The number of duplications ranged from 1 to 3 in the obese but metabolically healthy group, and from 2 to 11 in the obese and metabolically unhealthy group.

A comparative analysis of the patients without duplications/deletions and those with these genetic alterations revealed no statistically significant differences in their BMIs; adipose tissue percentages; or their adiponectin, leptin, insulin, or HOMA-IR levels. However, when comparing the patients without duplications in the *SEZ6L2-1* and *SH2B1-2* regions to those with duplications, significant differences were observed in their insulin levels (median: 16.8 vs. 34.10; *p* = 0.05; Mann–Whitney U test), HOMA-IR (median: 4.30 vs. 13.81; *p* = 0.03; Mann–Whitney U test), and trunk adipose tissue percentages (41.69 ± 5.62 vs. 36.12 ± 2.68; *p* = 0.056; one-way ANOVA). There were no significant differences regarding their lipidic profiles.

In the analysed cohort, the prevalence of clinical phenotypes was as follows: metabolically healthy obese, 25.42% (15 patients); metabolically unhealthy obese, 62.71% (37 patients); non-obese metabolically healthy, 3.39% (2 patients); and non-obese metabolically unhealthy, 8.47% (5 patients). The following duplications were detected in two of the obese metabolically healthy patients: one patient presented with a duplication of exon 3 in the *LEP* gene (07q32.1, 127.681.666-127.681.729), while the second patient showed duplications of exon 1 in the *SIM1* gene (06q16.3, 101.017.851-101.017.925), exon 2 in the *SH2B1* gene (16p11.2, 28.785.780-28.785.840), and exon 1 in the *SEZ6L2* gene (16p11.2, 29.817.845-29.817.905). The statistical analysis revealed a 1.2-fold higher relative risk for the patients with these duplications of being diagnosed as obese and metabolically healthy (*p* = 0.58) compared to the other clinical phenotypes. Regarding the patients in the obese and metabolically unhealthy group, five of them had duplications with or without concurrent deletions, with a 1.56-fold greater relative risk of being diagnosed as obese and metabolically healthy (*p* = 0.47) compared to the other clinical phenotypes. When considering the shared duplications of exon 2 in the *SH2B1* gene and exon 1 in the *SEZ6L2* gene, the relative risk for classification in the obese and metabolically unhealthy group was 1.85 times higher than in the other groups (*p* = 0.52). No duplications or deletions were reported in the non-obese patient groups, defined according to the BMI criteria.

## 3. Discussion

In the present study, duplications and/or deletions were identified in the genes involved in the physiopathological process of obesity in 11.86% of all study participants. All these patients exhibited adipose tissue percentages above the normal values for both males and females, though not all were classified as obese according to the BMI criteria. The aim of this study was to assess the prevalence of potential duplications and deletions in patients with excess adipose tissue and to analyse their implications for obesity profiles that have been documented in the literature.

No deletions or duplications were reported in the non-obese patients, defined according to their BMI, even when these individuals presented with adipose tissue levels exceeding the normal range. The mutation detection rate was similar between the two groups of patients with a BMI ≥ 30 kg/m^2^ (13.51% in obese, metabolically healthy vs. 13.33% in obese, metabolically unhealthy), with the prevalence of obese, metabolically healthy patients being lower. A significant difference that may be used to distinguish between groups with or without metabolic syndrome is the number of duplications, which is markedly higher in patients with metabolic syndrome. Deletions are only observed in patients with metabolic syndrome, although this group also includes patients without deletions, similar to the group without metabolic syndrome. Numerous studies, including meta-analyses of genome-wide association studies, have identified multiple loci associated with the adipose tissue percentage or directly with metabolic profiles, such as those located within or near the following genes: *FTO*, *IRS1*, *SPRY2*, *COBLL1*/*GRB14*, *IGF2BP1*, *PLA2G6*, and *CRTC1*. Additionally, several variants have been associated with increased adiposity but reduced cardiometabolic risk, characteristic of the obese, metabolically healthy group [6,9]. A recent study that followed obese metabolically healthy patients for over 15 years identified a statistically significant association between single-nucleotide polymorphisms and this obesity phenotype in women, specifically for the *FTO* (rs1121980), *TCF7L2* (rs7903146), and *SLC39A8* (rs13107325) genes [10]. In our study, the associations between the different clinical obesity phenotypes and the present mutations are not statistically significant, and the prevalence ratio values are considered small, given that this is an observational study [11].

Leptin receptor mutations have been associated with metabolic abnormalities, such as obesity, disruption of energy homeostasis, dyslipidaemia, and hyperglycaemia [12]. The binding of leptin to its receptor, which is expressed in specific brain regions, activates numerous biological processes, including neuroendocrine systems, adipose tissue function, autonomic functions, and the balance of insulin and glucose metabolism [13,14]. An *LEPR* deficiency is often underdiagnosed, with only 88 cases reported in the literature, of which 21 involved individuals of European descent. This contrasts with the estimated prevalence, which has suggested there are approximately 998 patients with an *LEPR* deficiency in Europe. The reported genetic alterations include single amino acid substitutions, insertions, duplications, and deletions [15,16]. Our study results highlight a partial deletion of the *LEPR* gene, involving exons 5, 9, and 14, which are located in the extracellular domain of the leptin receptor responsible for leptin binding [17]. These deletions may impair leptin recognition by the receptor, potentially influencing the circulating levels of this adipokine. To date, there has not been any report of a significant increase in leptin levels in patients with congenital leptin receptor deficiency. However, the available data from human studies are limited. In light of these preliminary findings, leptin cannot be used as a marker for this rare, autosomal recessive disorder [18,19]. In our study, the patient with a partial deletion of the *LEPR* gene had a leptin level of 13 ng/mL, which placed them within the lower third of the normal range for this biomarker. Although leptin secretion normally correlates with adipose tissue mass [20], we hypothesise that the relatively low leptin level in this patient, who had a fairly high adipose tissue mass, may have resulted from impaired leptin feedback due to the partial *LEPR* deletion, leading to dysfunctional leptin regulation.

In our study, we also analysed the 16p11.2 region, which includes the *SH2B1* and *SEZ6L2* genes, where duplications were identified. Insulin and leptin signalling are the primary pathways involved in *SH2B1*’s role in energy homeostasis. *SH2B1* stimulates the activity of Janus kinases (JAK1 and JAK2) and the formation of JAK2/insulin receptor substrate (*IRS1* and *IRS2*) complexes, promoting leptin signalling [21,22,23]. Additionally, *SH2B1* protects JAKs and IRSs from dephosphorylation [24,25], allowing them to act as effectors for growth hormone, insulin-like growth factor 1 (IGF-1), and nerve growth factor. Mutations within this gene can disrupt the signalling of these hormones [26]. The patients in our study who exhibited duplications in *SH2B1* also displayed duplications in the *SEZ6L2* gene. Therefore, the size of the duplication was identified in these patients at chromosomal band 16p11.2. Alterations in this region have been linked to obesity pathogenesis across multiple independent studies [27]. Furthermore, genetic variants in obese patients that influence insulin receptor signalling or relate to it have also been documented [6], supporting our findings of two-fold elevated insulin levels or three-fold increased HOMA-IR values in the subjects with the duplications mentioned above. Unlike many genetic studies in the field of obesity that rely solely on the BMI, this research stratified the participants by adipose tissue distribution and metabolic health. At the same time, while prior studies have focused on common obesity-associated SNPs or large CNVs, this study specifically investigated smaller CNVs in the genes involved in leptin signalling and insulin resistance. This revealed that *SH2B1-2*/*SEZ6L2-1* duplications are associated with reduced trunk fat despite higher insulin resistance, a paradoxical finding suggesting leptin pathway modulation. Moreover, whereas earlier studies often focused on extreme obesity or syndromic cases [28,29,30], this study identified small CNVs in a cohort of seemingly healthy individuals, but with high adipose tissue percentages. This highlights CNVs as contributors to subtle metabolic dysfunction even in non-syndromic obesity. In the future, genetic profiling including these modifications may be able to predict which obese patients are prone to metabolic dysfunction despite a “favorable” fat distribution.

While a deletion at the chromosomal band 16p11.2 level is linked to an increased BMI and obesity risk [31,32], a duplication is associated with underweight and low BMI [33]. Consequently, the severe obesity associated with deletions and the underweight linked to duplications might have a mirror-like aetiology, possibly due to the contrasting effects on energy balance [33,34]. Furthermore, the variability in the duplications in this region is high, suggesting a significant contribution from familial and additional genetic factors [34]. Our results show similar BMI values between the two groups. However, the mean percentage of trunk adipose tissue is lower in the patients with duplications of the *SH2B1* and *SEZ6L2* gene exons, with statistical significance. Considering the studies mentioned earlier, we hypothesise a relationship between the studied region and abdominal adipose tissue as a parameter of body composition, rather than of BMI.

A key aspect to consider is that ethnic variability may influence the CNVs–phenotype association. For instance, Windholz J. et al. performed an MLPA analysis on a cohort of 194 obese Caucasian children and reported no CNVs in the *POMC*, *LEP*, *LEPR*, *MC4R*, *MC3R*, and *MC2R* genes related to obesity [28]. At the same time, D’Angelo C.S. et al. identified, among a cohort of 338 Brazilian patients with syndromic obesity, 18 deletions and five duplications of specific loci in regions associated with obesity, among which 16p11.2 was included [29]. Further research on the same group pointed out a recurrent 600 kb 16p11.2 proximal deletion in one patient and also a duplication of the same genomic rearrangement in another patient, who both presented with obesity [30]. Genomic deletions in the 16p11.2 region have also been reported in non-syndromic obesity, and they were often of de novo origin since there were known differences in the flanking duplication patterns. Da Silva Assis highlighted the differences in adipokine-related CNVs between Brazilian and European populations, underscoring the need for ethnically diverse studies [35]. Our findings align partially with these reports, but also introduce novel CNVs–biomarker relationships, particularly regarding leptin, adiponectin, and insulin levels. Mutations in the *SIM1* gene are a well-documented cause of monogenic obesity. There have been reported cases of Prader–Willi syndrome and hypopituitarism in patients with chromosomal deletions containing the *SIM1* gene [36,37]. Disruptions in the leptin–melanocortin–oxytocin pathway [38] or alterations in energy balance regulation [39] can be consequences of *SIM1* mutations contributing to the development of obesity. In our study, we also identified a partial deletion of the *SIM1* gene (exon 7 deletion), alongside the deletion of exon 3 of the *LEP* gene and exon 1 of the *MC3R* gene, all present in the same patient.

This study has several limitations. Firstly, the sample size may limit the generalizability of our findings, particularly for the rare CNVs, like the partial *LEPR* deletion observed in only one patient in our study. We acknowledge the need for larger cohorts to also validate the prevalence and effect size of the statistically significant association of *SH2B1-1*/*SEZ6L2-1* duplication with metabolic dysfunction. We also acknowledge the need for functional studies to clarify various mechanisms, like the role of *SEZ6L2* in leptin signalling. Secondly, the cross-sectional design of this study can only determine if the observed CNVs are associated with metabolic dysfunction. In order to establish a possible causal relationship, longitudinal or interventional studies are needed. However, our work provides a framework for integrating CNV data into existing genetic risk scores for obesity, since this research focused on identifying which exons in particular genes could provide a more precise genetic profile. While our findings are not immediately translatable to clinical practice, they contribute to the development of a more comprehensive CNV database that could ultimately be used to produce algorithms for early obesity risk stratification, metabolic risk assessment, and personalised intervention strategies targeting specific genomic regions. Moreover, the MLPA technique employed in this study offers a practical, rapid, and cost-effective approach for initial screening, proving its potential utility in obesity-related genetic investigation.

Both genetic counselling and precise knowledge about the mutation in a specific patient will enable the future application of personalised genetic therapies. The MLPA technique used in this study facilitates the easy identification of aberrant copy numbers of up to 60 specific nucleic acid sequences through a simple PCR reaction. As a result, the time required for mutation screening of one or more genes is significantly reduced. The current research highlights the importance of accurately identifying genetic alterations, particularly in obesity, which could be used in future therapies. For Duchenne muscular dystrophy, there is already an exon-skipping therapy, which requires defining the exact endpoints of a large deletion or duplication or the precise identification of the exon with a mutation in order to enable the removal of the affected region of the gene [40,41,42]. In the field of adiposity, significant progress is being made in genomic editing using CRISPR technology. The team of Matharu et al. successfully targeted the non-coding genomic regions of *SIM1* and *MC4R* using CRISPR technology, alleviating obesity syndrome caused by haploinsufficiency in a murine model [43].

## 4. Materials and Methods

The number of subjects enrolled in this research was reduced from 474 consecutive patients presenting to a regional hospital-based Cardiology Department for evaluation, to 59 patients who met this study’s inclusion and exclusion criteria. All subjects were between 35 and 75 years of age and exhibited a body fat percentage above the normal threshold (>25% for men and >35% for women), with body mass index (BMI) greater than 25 kg/m^2^, although not all surpassed the diagnostic threshold for obesity as defined by BMI (30 kg/m^2^). The aim was to include subjects classified as obese based on adipose tissue percentage, as there is compelling evidence that the quantity of adipose tissue is a more significant indicator of metabolic health than BMI [44,45,46].

This observational, cross-sectional study was performed over a period of 2 years and was approved by the University Ethics Committee. All participants agreed to and signed an informed consent prior to entering this study. The subjects enrolled in this study did not have a history of acute atherosclerotic conditions (acute myocardial infarction, acute peripheral arterial disease, or acute stroke), and had not been diagnosed with a chronic disease or received treatment for a chronic condition in the last 6 months. Therefore, the selected cohort were seemingly healthy individuals who exhibited high percentages of adipose tissue. However, during evaluation we identified subjects with underdiagnosed metabolic syndrome; therefore, four clinical phenotypes were described: metabolically healthy obese, metabolically unhealthy obese, metabolically healthy non-obese, and metabolically unhealthy non-obese. Obesity was defined by BMI ≥ 30 kg/m^2^ and unhealthy status by the presence of metabolic syndrome: waist circumference > 88 cm/104cm (women/men) and at least 2 of the following criteria—glucose ≥ 100 mg/dL, HDL-C < 40 mg/dL (men)/< 50 mg/dL (women), TG ≥ 150 mg/dL, and TAS/TAD ≥ 130/85 mmHg [47].

Whole-body composition was quantified using dual-energy X-ray Absorptiometry (DEXA—Hologic QDR Delphy A fan-beam densitometer, Hologic Inc., MA). The biochemical analysis for each participant included enzyme-linked immunosorbent assay (ELISA) for adiponectin and leptin levels; chemiluminescence assay for insulin; and spectrophotometric methods for glucose, total cholesterol, LDL cholesterol, HDL cholesterol, and triglycerides. The CRP measurement was based on latex-enhanced turbidimetry. Insulin resistance was assessed using the homeostasis model assessment (HOMA-IR) index, calculated according to Matthews et al. [48], using the following equation: HOMA-IR = [insulin (μU/mL) × glucose (mg/dL)]/405.

**Figure 5:**
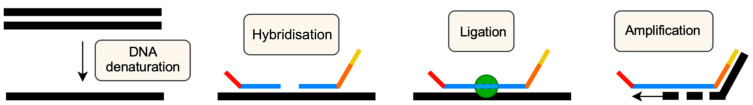
MLPA technique (adapted from [49]).

To identify potential deletions or duplications in genes associated with obesity, the MLPA technique was employed. The kit used was a Salsa^®^ MLPA^®^ Probemix P220-B3 Obesity (MRC-Holland, Amsterdam, The Netherlands), which contains 47 MLPA probes with amplified products ranging from 130 to 495 nucleotides. This includes 4 probes for 16p11.2 region (including genes *SH2B1* and *SEZ6L2*), 11 probes for *LEPR*, 4 probes for *POMC*, 8 probes for *SIM1*, 4 probes for genes flanking *SIM1*, 3 probes for *LEP*, 2 probes for *MC4R*, 1 probe for *MC2R*, and 2 probes for *MC3R*. Eight reference probes were included to detect autosomal chromosomal locations. The results were analysed using Coffalyser.net software, version v.140721.1958, and capillary electrophoresis was conducted with an Applied Biosystems ABI Hitachi 3500 Genetic Analyzer (Thermo Fisher Scientific, USA, Hitachi High-Technologies, Japan). The principle of the MLPA technique is illustrated in Figure 5. Blood samples were collected in vacutainers with EDTA-K_3_ and immediately transported to the laboratory for DNA extraction using a Wizard^®^ Genomic DNA Purification Kit (Promega Corporation, Madison, WI, USA). DNA was then denaturated by heating for 5 min in a thermal cycler, followed by the addition of the MLPA probe mix. Each MLPA probe consisted of two oligonucleotides, which hybridised overnight to adjacent target DNA sequences. This was followed by ligation using a specific ligase enzyme, which was subsequently deactivated by heating. During the PCR (Polymerase Chain Reaction) process, all probes were amplified simultaneously using the same PCR prime pair. The distinct nucleotide count for each probe ensured its uniqueness. The forward PCR primer, labelled with a fluorescent marker, enabled visualisation of the amplified products during capillary electrophoresis. The resulting electrophoregram was analysed with Coffalyser.net (version v.140721.1958), which normalised the data by comparing each sample to a reference set and calculated a ratio for each MLPA probe in each patient sample. The analysis of these ratios allowed for the detection of duplications or deletions.

Statistical analysis was performed with Microsoft Excel v.16.64 (Microsoft Corporation, Washington, DC, USA) and SPSS v.23.0 (IBM Corporation, Armonk, NY, USA). Kolmogorov–Smirnov test and histogram visual analysis were performed for assessing normality. For comparative analysis between continuous variables, Mann–Whitney U test was applied as the non-parametric test and one-Way ANOVA as the parametric test. Normally distributed data were reported as mean ± standard deviation and non-normally distributed data as median, along with quartiles. Nominal variables were presented as frequencies and percentages. The statistically significant threshold was considered *p* < 0.05.

## 5. Conclusions

The interpretation of genetic modifications is difficult and complex. However, identifying the genetic predisposition to a specific metabolic dysregulation pattern will eventually enable personalised medical interventions for prevention and treatment. Obesity is directly linked to metabolic syndrome and has a prevalence that increases with age [50]. Therefore, the screening panel used in this study is best applied at a young age, when it could significantly contribute to assessing a patient’s risk of developing obesity in the future, and consequently, cardiometabolic pathologies. This outcome could facilitate the mapping of personalised interventions to possibly modulate phenotypic expression.

## Figures and Tables

**Table 1:** Demographic, anthropometric, and biochemical data for this study’s cohort.

Parameter	Frequency	Percent	
Residence			
Urban	37	62.7%	
Rural	22	37.3%	
Gender			
Female	42	71.2%	
Male	17	28.8%	
Current smoker			
Yes	8	13.6%	
No	51	86.4%	
**Parameter**	**Median (Q3–Q1)**	**Min**	**Max**
Age	58 (50–64)	39	72
Adiponectin	12.30 (14.40–9.24)	4.53	28.53
Leptin	29.10 (42.40–14)	3.10	58.20
Insulin	17.30 (29.80–11.10)	3.72	51.10
Glucose	101 (94–123)	78	242
HOMA-IR	4.41 (8.94–2.89)	0.81	27.25
Total cholesterol	196 (230–166)	108	351
LDL cholesterol	124 (151–102.2)	36.2	266
Triglycerides	170.58 (233–102)	57	488
CRP	0.32 (0.16–0.53)	0.04	2.51
**Parameter**	**Mean ± S.D.**	**C.I. for mean (95%)**	
		**Lower bound**	**Upper bound**
BMI	34.25 ± 4.67	33.03	35.47
Total adipose tissue (%)	40.70 ± 5.81	39.19	42.21
Trunk adipose tissue (%)	41.31 ± 5.64	39.85	42.79
HDL cholesterol	50.07 ± 14.08	46.40	53.74

Note: LDL = low-density lipoprotein; HDL = high-density lipoprotein; CRP = C-reactive protein.

**Table 2:** Clinical and molecular characteristics of subjects with duplications/deletions.

	Subject No.	4	45	47	53	27	52	43
Parameter								
**General characteristics**								
Gender		Female	Male	Male	Female	Female	Female	Female
Age		52	71	61	56	49	63	63
BMI (kg/m^2^)		32.97	36.52	35.34	34.30	35.70	34.20	39.27
Total adipose tissue (%)		39.8	31.9	31.1	43.5	50.3	40.6	41.5
Trunk adipose tissue (%)		40	34.1	33.6	37.8	50	39	41.1
Adiponectin (μg/dL)		9.24	9.72	11.22	17.64	5.85	13.26	17.82
Leptin (ng/dL)		19.87	18.7	13.9	46.8	44.29	22.3	13
Insulin (μUI/mL)		15.9	51.1	32.4	15.9	12.2	35.8	6.76
Glucose (mg/dL)		94	156	175	95	98	154	109
HOMA-IR		3.69	19.68	14	3.73	2.95	13.61	1.82
Total cholesterol (mg/dL)		240	138	188	237	143	242	298
LDL cholesterol (mg/dL)		151	85	94	151	89	204	205
HDL cholesterol (mg/dL)		82	27	20	76	30	50	59
Triglycerides (mg/dL)		108	227	432	85	161	152	271
CRP (mg/dL)		0.29	0.29	0.20	0.29	0.40	1.22	1.75
**Genetic parameters**								
**No. of duplications**		** 1 **	** 2 **	** 2 **	** 3 **	** 11 **	** 3 **	** 3 **
**No. of deletions**		** 0 **	** 0 **	** 0 **	** 0 **	** 0 **	** 3 **	** 4 **
* LEPR *						Dupl(7) ^e^ Dupl(11) ^f^ Dupl(14) ^g^ Dupl(16) ^h^ Dupl(18) ^i^ Dupl(20) ^j^ Dupl(23) ^k^		Del(5) ^q^ Del(9) ^r^ Del(14) ^g^
* LEP *		Dupl(3) ^a^				Dupl(2) ^l^	Del(3) ^a^	Dupl(3) ^a^
* SIM1 *					Dupl(1) ^d^	Dupl(7) ^m^	Dupl(1) ^d^ Del(7) ^m^	Dupl(2) ^s^
* POMC *								Dupl(4) ^t^
* COQ3 *						Dupl(5) ^n^		
*GRIK2*		-						
*NR2E1*		-						
* CDK19 *								Del(6) ^u^
* SH2B1 *			Dupl(2) ^b^	Dupl(2) ^b^	Dupl(2) ^b^		Dupl(2) ^b^	
* SEZ6L2 *			Dupl(1) ^c^	Dupl(1) ^c^	Dupl(1) ^c^		Dupl(1) ^c^	
* MC4R *						Dupl(1) ^o^		
* MC3R *							Del(1) ^p^	
* MC2R *		-						

Note 1: LDL = low-density lipoprotein; HDL = high-density lipoprotein, CRP = C-reactive protein. Note 2: dupl= duplication; del = deletion; exons are indicated in parentheses. Chromosomal band notation and genomic coordinates (hg18 assembly) are as follows: ^a^ 07q32.1–127681666-127681729; ^b^ 16p11.2–28785780-28785840; ^c^ 16p11.2–29817845-29817905; ^d^ 06q16.3–101017851-101017925; ^e^. 01p31.3–65831011-65831083; ^f^ 01p31.3–65840155-65840234; ^g^ 01p31.3–65848260-65848323; ^h^ 01p31.3–65854392-65854458; ^i^ 01p31.3–65858191-65858268; ^j^ 01p31.3–65861193-65861264; ^k^ 01p31.3–65872115-65872186; ^l^ 07q32.1–127679400-127679466; ^m^ 06q16.3–101002734-101002793; ^n^ 06q16.3–99930640-99930718; ^o^ 18q21.32–56190649-56190722; ^p^ 20q13.2–54258130-54258193; ^q^ 01p31.3–65808957-65809025; ^r^ 01p31.3–65836957-658387026; ^s^ 06q16.3–101008344-101008400; ^t^ 02p23.3–25237284-25237359; and ^u^ 06q21–111059985-111060057.

## Data Availability

Data is contained within the article.

## Supplementary Materials

The following supporting information can be downloaded at: https://www.mdpi.com/article/10.3390/ijms26104782/s1.
