# Supplementary material for: Towards Precision Medicine in Obesity: Genetic Copy Number Variations Profiling Linked to Specific Metabolic Dysregulation Patterns
Source: Int J Mol Sci. 2025 May 16;26(10):4782. doi: 10.3390/ijms26104782 (PMC12112116; doi:10.3390/ijms26104782)
Supplement: Supplementary file 1 [file ijms-26-04782-s001.zip › figures/fig S3.pdf]

COFFALYSER

# Sample report: 45

*Sample type: Sample | Project: 20220727 | Experiment: 20220727 tura 2 | Dye: 6-FAM | Performed by: Admin*
*Machine: ABI-3500 | Report date: 7/27/2022 | Run date: 7/27/2022 | Software Version: v.140721.1958 | Normal range: 0.7 - 1.3*

| Authorization | |
|---|---|
| Date | |

| | | | | | |
|---|---|---|---|---|---|
| **MLPA probe mix:** | P220-Obesity | **Nr of test probes:** | 47/47 | **FRSS:** | OK 100% |
| **Lot number:** | B3-0919 | **Nr of ref probes** | 8/8 | **FRMS:** | OK 100% |
| **Sheet date:** | 5/31/2022 9:45:02 AM | **DNA concentration:** | OK | **PSLP:** | OK -9% |
| **Control fragments:** | CF-003-[brown] QDX2 (A2-1 | **DNA denaturation** | OK | **RSQ:** | OK |
| **Analysis method:** | Block SSC: On | **Expected gender:** | Male | **RPQ:** | Warning |
| **Used metric:** | Peak height | **Residual primer %** | OK 13% | **CAS:** | OK? 85% |

**Reference Samples:** C | I | O

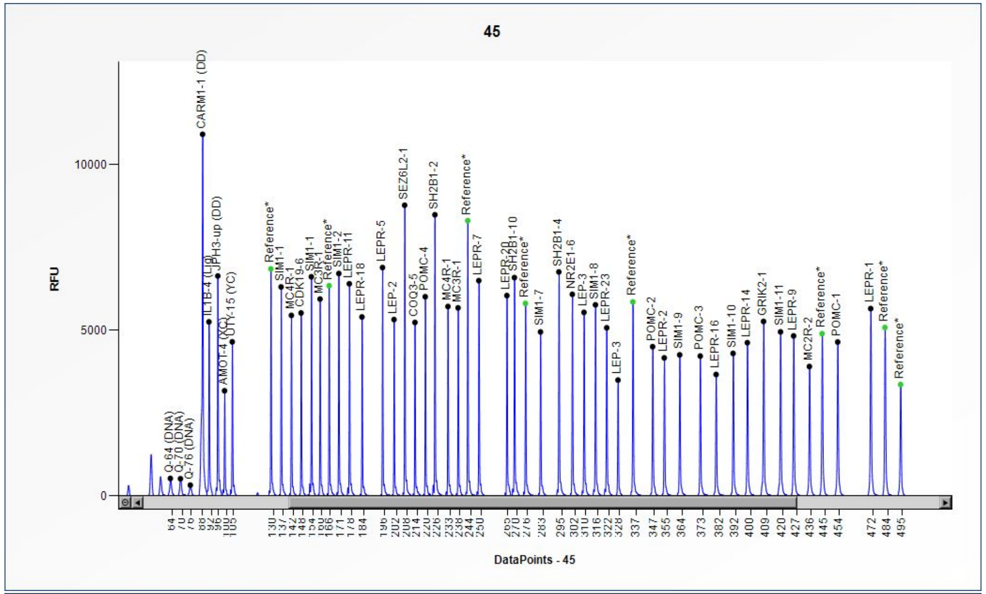


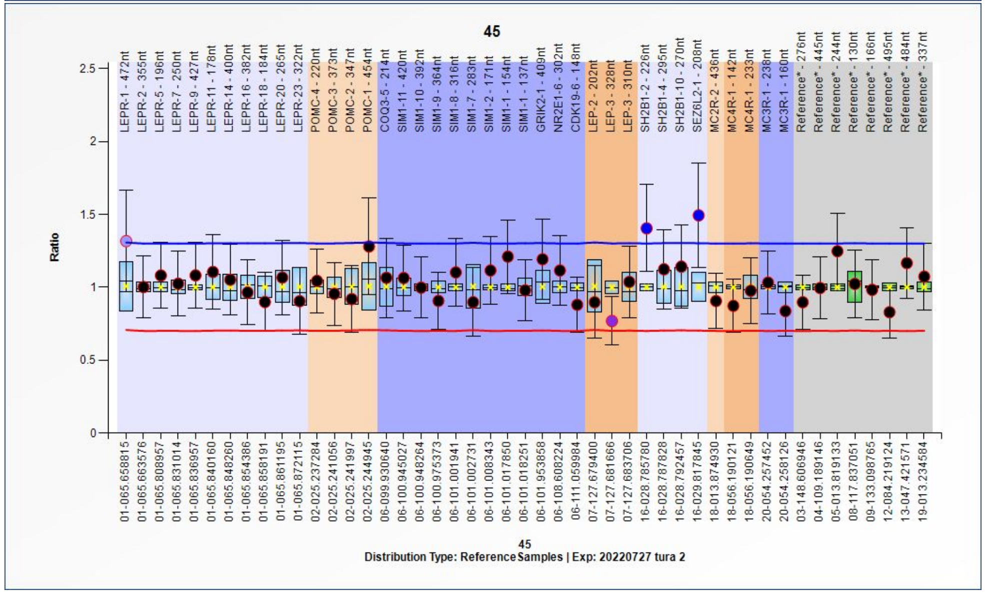


| D [nt] | Gene-Exon | Chr.band | hg18 loc. | Height | Area | Ratio[H] | Stdev | [REF] | [Sam] | Width | d[nt] |
|---|---|---|---|---|---|---|---|---|---|---|---|
| 472 | LEPR-1 | 01p31.3 | 01-065.658815 | 5658 | 42438 | **1.32** | **0.17** | >* | ? | 63 | 0.2 |
| 355 | LEPR-2 | 01p31.3 | 01-065.663576 | 4172 | 25361 | *1* | **0.11** | = | = | 59 | 0.0 |
| 196 | LEPR-5 | 01p31.3 | 01-065.808957 | 6898 | 30562 | *1.08* | **0.11** | = | = | 42 | 0.0 |
| 250 | LEPR-7 | 01p31.3 | 01-065.831014 | 6503 | 31682 | *1.02* | **0.11** | = | = | 46 | 0.1 |
| 427 | LEPR-9 | 01p31.3 | 01-065.836957 | 4835 | 33789 | *1.08* | **0.11** | = | = | 63 | 0.1 |
| 178 | LEPR-11 | 01p31.3 | 01-065.840160 | 6408 | 29690 | *1.11* | **0.13** | = | = | 47 | 0.1 |
| 400 | LEPR-14 | 01p31.3 | 01-065.848260 | 4630 | 30908 | *1.05* | **0.12** | = | = | 60 | 0.1 |
| 382 | LEPR-16 | 01p31.3 | 01-065.854386 | 3670 | 24031 | *0.96* | **0.11** | = | = | 70 | 0.0 |
| 184 | LEPR-18 | 01p31.3 | 01-065.858191 | 5406 | 23733 | *0.9* | 0.1 | = | = | 50 | 0.1 |
| 265 | LEPR-20 | 01p31.3 | 01-065.861195 | 6052 | 29056 | *1.07* | **0.13** | = | = | 36 | 0.0 |
| 322 | LEPR-23 | 01p31.3 | 01-065.872115 | 5080 | 30207 | *0.9* | **0.11** | = | = | 52 | 0.0 |
| 220 | POMC-4 | 02p23.3 | 02-025.237284 | 6017 | 27144 | *1.04* | **0.11** | = | = | 44 | 0.0 |
| 373 | POMC-3 | 02p23.3 | 02-025.241056 | 4226 | 26829 | *0.95* | **0.11** | = | = | 61 | 0.0 |
| 347 | POMC-2 | 02p23.3 | 02-025.241997 | 4510 | 27264 | *0.92* | **0.11** | = | = | 58 | 0.1 |
| 454 | POMC-1 | 02p23.3 | 02-025.244945 | 4651 | 34035 | *1.28* | **0.17** | = | = | 71 | 0.1 |
| 214 | COQ3-5 | 06q16.3 | 06-099.930640 | 5240 | 24331 | *1.06* | **0.14** | = | = | 46 | 0.0 |
| 420 | SIM1-11 | 06q16.3 | 06-100.945027 | 4957 | 35639 | *1.06* | **0.11** | = | = | 64 | 0.0 |
| 392 | SIM1-10 | 06q16.3 | 06-100.948264 | 4306 | 28768 | *1* | **0.11** | = | = | 64 | 0.1 |
| 364 | SIM1-9 | 06q16.3 | 06-100.975373 | 4263 | 26617 | *0.91* | 0.1 | = | = | 63 | 0.0 |
| 316 | SIM1-8 | 06q16.3 | 06-101.001941 | 5772 | 33027 | *1.1* | **0.12** | = | = | 53 | 0.0 |
| 283 | SIM1-7 | 06q16.3 | 06-101.002731 | 4956 | 26232 | *0.9* | **0.12** | = | = | 49 | 0.0 |
| 171 | SIM1-2 | 06q16.3 | 06-101.008343 | 6718 | 31401 | *1.12* | **0.12** | = | = | 51 | 0.0 |
| 154 | SIM1-1 | 06q16.3 | 06-101.017850 | 6620 | 30584 | *1.21* | **0.13** | = | = | 39 | 0.0 |
| 137 | SIM1-1 | 06q16.3 | 06-101.018251 | 6312 | 28913 | *0.98* | 0.1 | = | = | 46 | 0.0 |
| 409 | GRIK2-1 | 06q16.3 | 06-101.953858 | 5271 | 37755 | *1.19* | **0.14** | = | = | 78 | 0.1 |
| 302 | NR2E1-6 | 06q21 | 06-108.608224 | 6089 | 32683 | *1.12* | **0.12** | = | = | 57 | -0.1 |
| 148 | CDK19-6 | 06q21 | 06-111.059984 | 5527 | 24648 | *0.88* | 0.09 | = | = | 40 | 0.0 |
| 202 | LEP-2 | 07q32.1 | 07-127.679400 | 5328 | 23128 | *0.9* | **0.12** | = | = | 47 | 0.0 |
| 328 | LEP-3 | 07q32.1 | 07-127.681666 | 3501 | 19328 | *0.77* | 0.08 | << | = | 40 | 0.0 |
| 310 | LEP-3 | 07q32.1 | 07-127.683706 | 5546 | 30696 | *1.04* | **0.12** | = | = | 42 | 0.0 |
| 226 | SH2B1-2 | 16p11.2 | 16-028.785780 | 8490 | 41220 | ***1.4*** | **0.15** | >>* | ? | 53 | 0.0 |
| 295 | SH2B1-4 | 16p11.2 | 16-028.787828 | 6765 | 36982 | *1.12* | **0.14** | = | = | 60 | 0.0 |
| 270 | SH2B1-10 | 16p11.2 | 16-028.792457 | 6595 | 33981 | *1.14* | **0.14** | = | = | 49 | 0.0 |
| 208 | SEZ6L2-1 | 16p11.2 | 16-029.817845 | 8777 | 39586 | ***1.49*** | **0.18** | >>* | ? | 50 | 0.0 |
| 436 | MC2R-2 | 18p11.21 | 18-013.874930 | 3912 | 28220 | *0.91* | 0.09 | = | = | 61 | 0.1 |
| 142 | MC4R-1 | 18q21.32 | 18-056.190121 | 5454 | 25271 | *0.87* | 0.09 | = | = | 47 | 0.0 |
| 233 | MC4R-1 | 18q21.32 | 18-056.190649 | 5722 | 27439 | *0.98* | **0.11** | = | = | 50 | 0.0 |
| 238 | MC3R-1 | 20q13.2 | 20-054.257452 | 5681 | 26650 | *1.03* | **0.11** | = | = | 43 | 0.1 |
| 160 | MC3R-1 | 20q13.2 | 20-054.258126 | 5948 | 26516 | *0.84* | 0.09 | = | = | 35 | 0.0 |
| 276 | Reference* | 03q24 | 03-148.606946 | 5815 | 29767 | *0.9* | 0.09 | = | = | 46 | 0.0 |
| 445 | Reference* | 04q25 | 04-109.189146 | 4903 | 35600 | *0.99* | **0.11** | = | = | 62 | 0.1 |
| 244 | Reference* | 05p15.2 | 05-013.819133 | 8312 | 39336 | *1.25* | **0.13** | = | = | 55 | 0.0 |
| 130 | Reference* | 08q24.11 | 08-117.837051 | 6857 | 32400 | *1.02* | **0.12** | = | = | 49 | 0.1 |
| 166 | Reference* | 09q34.13 | 09-133.098765 | 6349 | 29585 | *0.98* | 0.1 | = | = | 55 | -0.1 |
| 495 | Reference* | 12q21.31 | 12-084.219124 | 3367 | 25208 | *0.83* | 0.09 | = | = | 55 | 0.0 |
| 484 | Reference* | 13q14.2 | 13-047.421571 | 5088 | 37788 | *1.17* | **0.12** | = | = | 61 | 0.1 |
| 337 | Reference* | 19p13.13 | 19-013.234584 | 5862 | 35227 | *1.07* | **0.12** | = | = | 71 | 0.1 |
| **Median value all probe values:** | | | | **5546** | **29767** | **1.03** | **0.11*** | | | **52** | **0.01** |

Normal range: 0.7 - 1.3 Essential information on the use of this product is present in the product description which is available on http://www.mlpa.com. For questions, mailto:info@mlpa.com.
The 148 nt probe 03945-L03403, 214 nt probe 16153-L19387, 302 nt probe 03939-L09973, 409 nt probe 16154-L18338 are a "flanking probe". It is included to facilitate the determination of the extent of a deletion/duplication. Copy number alterations of flanking and reference probes are unlikely to be related to the condition tested. *** The signal of the 238 nt probe 08884-L08940 might be influenced by SNP rs61736060 (non-validated). In case of apparent deletions, it is recommended to sequence the region targeted by this probe.... Please see the product description for all notifications.
