# Supplementary material for: Towards Precision Medicine in Obesity: Genetic Copy Number Variations Profiling Linked to Specific Metabolic Dysregulation Patterns
Source: Int J Mol Sci. 2025 May 16;26(10):4782. doi: 10.3390/ijms26104782 (PMC12112116; doi:10.3390/ijms26104782)
Supplement: Supplementary file 1 [file ijms-26-04782-s001.zip › figures/fig S2.pdf]

COFFALYSER

Sample report: 43

Sample type: Sample | Project: 20220727 | Experiment: 20220727 tura 2 | Dye: 6-FAM | Performed by: Admin
Machine: ABI-3500 | Report date: 7/27/2022 | Run date: 7/27/2022 | Software Version: v.140721.1958 | Normal range: 0.7 - 1.3

Authorization	
Date	

**MLPA probe mix:**	P220-Obesity	**Nr of test probes:**	47/47	**FRSS:**	OK 100%
**Lot number:**	B3-0919	**Nr of ref probes**	8/8	**FRMS:**	Bad 40%
**Sheet date:**	5/31/2022 9:45:02 AM	**DNA concentration:**	OK	**PSLP:**	OK 1%
**Control fragments:**	CF-003-[brown] QDX2 (A2-1	**DNA denaturation**	Bad	**RSQ:**	Warning
**Analysis method:**	Block SSC: On	**Expected gender:**	Female	**RPQ:**	Bad
**Used metric:**	Peak height	**Residual primer %**	OK 9%	**CAS:**	Bad 10%

Reference Samples: C | I | O

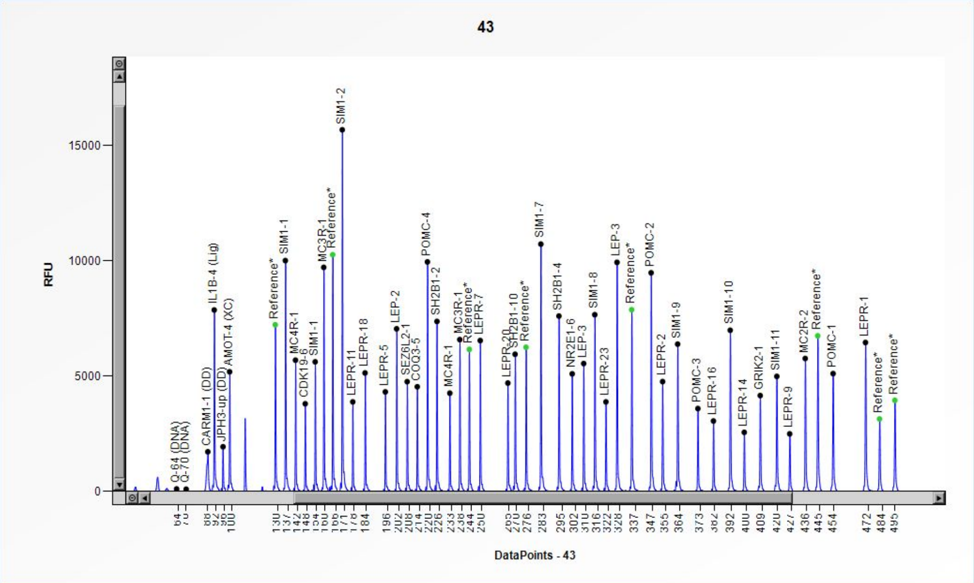

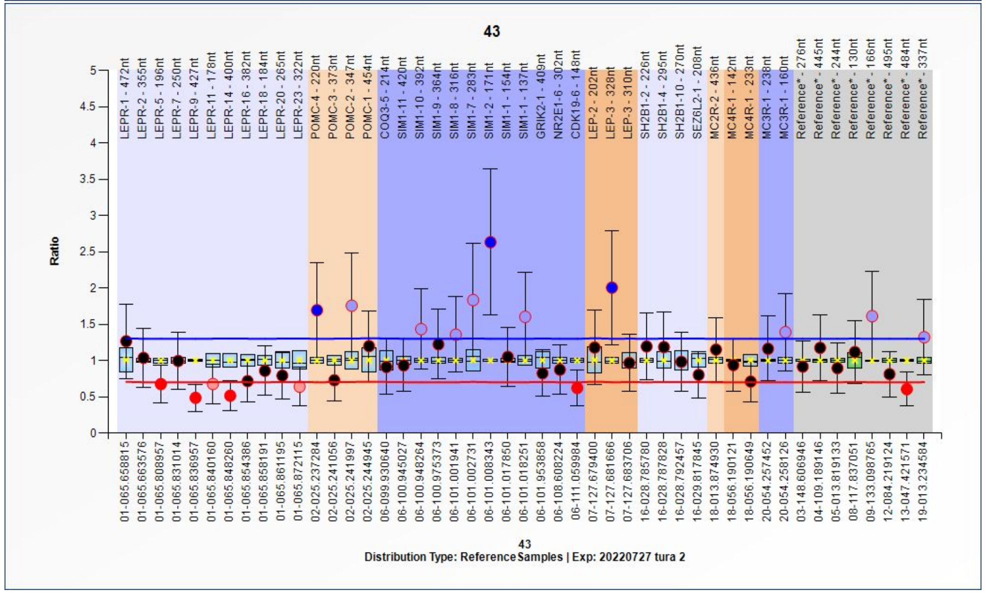

D [nt]	Gene-Exon	Chr.band	hg18 loc.	Height	Area	Ratio[H]	Stdev	[REF]	[Sam]	Width	d[nt]
472	LEPR-1	01p31.3	01-065.658815	6463	42831	1.27	0.26	=	=	72	0.2
355	LEPR-2	01p31.3	01-065.663576	4767	26488	1.04	0.2	=	=	57	0.0
196	LEPR-5	01p31.3	01-065.808957	4323	19271	0.67	0.13	<<*	<*	51	0.1
250	LEPR-7	01p31.3	01-065.831014	6546	30488	0.99	0.19	=	=	46	0.0
427	LEPR-9	01p31.3	01-065.836957	2503	15133	0.49	0.09	<<*	<<*	41	0.2
178	LEPR-11	01p31.3	01-065.840160	3889	17821	0.68	0.14	<*	<*	38	0.0
400	LEPR-14	01p31.3	01-065.848260	2570	15425	0.51	0.1	<<*	<<*	51	0.0
382	LEPR-16	01p31.3	01-065.854386	3061	17319	0.72	0.14	=	=	41	0.0
184	LEPR-18	01p31.3	01-065.858191	5143	24446	0.86	0.17	=	=	65	0.1
265	LEPR-20	01p31.3	01-065.861195	4704	22891	0.79	0.16	=	=	37	0.1
322	LEPR-23	01p31.3	01-065.872115	3888	20236	0.64	0.13	<*	<*	51	0.1
220	POMC-4	02p23.3	02-025.237284	9956	45894	1.7	0.32	>>*	>*	48	0.0
373	POMC-3	02p23.3	02-025.241056	3601	20265	0.73	0.14	=	=	54	0.0
347	POMC-2	02p23.3	02-025.241997	9484	51307	1.76	0.36	>*	>*	66	0.1
454	POMC-1	02p23.3	02-025.244945	5115	32906	1.2	0.24	=	=	69	0.1
214	COQ3-5	06q16.3	06-099.930640	4546	20583	0.91	0.19	=	=	43	0.0
420	SIM1-11	06q16.3	06-100.945027	4994	31093	0.93	0.18	=	=	48	0.1
392	SIM1-10	06q16.3	06-100.948264	6994	40742	1.43	0.28	>*	>*	66	0.1
364	SIM1-9	06q16.3	06-100.975373	6392	37844	1.22	0.24	=	=	70	0.1
316	SIM1-8	06q16.3	06-101.001941	7668	39735	1.36	0.26	>*	?	56	0.1
283	SIM1-7	06q16.3	06-101.002731	10726	53326	1.83	0.39	>*	>*	54	0.1
171	SIM1-2	06q16.3	06-101.008343	15679	70975	2.63	0.51	>>*	>>*	52	0.0
154	SIM1-1	06q16.3	06-101.017850	5625	25826	1.05	0.2	=	=	34	0.1
137	SIM1-1	06q16.3	06-101.018251	10013	47442	1.6	0.31	>*	>*	48	0.1
409	GRIK2-1	06q16.3	06-101.953858	4163	25352	0.82	0.16	=	=	61	0.0
302	NR2E1-6	06q21	06-108.608224	5104	26335	0.87	0.17	=	=	57	-0.1
148	CDK19-6	06q21	06-111.059984	3807	17914	0.62	0.12	<<*	<*	41	0.0
202	LEP-2	07q32.1	07-127.679400	7055	31695	1.18	0.26	=	=	50	0.1
328	LEP-3	07q32.1	07-127.681666	9937	51162	2.01	0.39	>>*	>*	54	0.1
310	LEP-3	07q32.1	07-127.683706	5550	28525	0.97	0.2	=	=	49	0.0
226	SH2B1-2	16p11.2	16-028.785780	7377	35165	1.19	0.23	=	=	52	-0.1
295	SH2B1-4	16p11.2	16-028.787828	7609	38359	1.19	0.24	=	=	63	0.0
270	SH2B1-10	16p11.2	16-028.792457	5953	29933	0.98	0.2	=	=	39	0.1
208	SEZ6L2-1	16p11.2	16-029.817845	4765	22583	0.8	0.16	=	=	50	0.1
436	MC2R-2	18p11.21	18-013.874930	5770	35512	1.15	0.22	=	=	63	0.1
142	MC4R-1	18q21.32	18-056.190121	5696	25800	0.94	0.18	=	=	49	0.1
233	MC4R-1	18q21.32	18-056.190649	4267	20180	0.71	0.14	=	=	44	-0.1
238	MC3R-1	20q13.2	20-054.257452	6589	30321	1.16	0.22	=	=	42	0.0
160	MC3R-1	20q13.2	20-054.258126	9718	44536	1.39	0.27	>*	>*	36	0.1
276	Reference*	03q24	03-148.606946	6256	31615	0.92	0.18	=	=	65	0.1
445	Reference*	04q25	04-109.189146	6751	42188	1.17	0.23	=	=	53	0.2
244	Reference*	05p15.2	05-013.819133	6163	29131	0.89	0.17	=	=	44	-0.1
130	Reference*	08q24.11	08-117.837051	7230	35734	1.12	0.22	=	=	48	0.1
166	Reference*	09q34.13	09-133.098765	10268	44796	1.61	0.31	>*	>*	40	0.0
495	Reference*	12q21.31	12-084.219124	3957	26336	0.81	0.16	=	=	52	-0.1
484	Reference*	13q14.2	13-047.421571	3148	20836	0.6	0.12	<<*	<*	72	0.1
337	Reference*	19p13.13	19-013.234584	7882	42824	1.32	0.26	>*	?	65	0.1
**Median value all probe values:**				**5770**	**30321**	**0.99**	**0.2***			**51**	**0.07**

Normal range: 0.7 - 1.3 Essential information on the use of this product is present in the product description which is available on http://www.mlpa.com. For questions, mailto:info@mlpa.com.
The 148 nt probe 03945-L03403, 214 nt probe 16153-L19387, 302 nt probe 03939-L09973, 409 nt probe 16154-L18338 are a “flanking probe”. It is included to facilitate the determination of the extent of a deletion/duplication. Copy number alterations of flanking and reference probes are unlikely to be related to the condition tested. *** The signal of the 238 nt probe 08884-L08940 might be influenced by SNP rs61736060 (non-validated). In case of apparent deletions, it is recommended to sequence the region targeted by this probe.... Please see the product description for all notifications.
