# Supplementary material for: Towards Precision Medicine in Obesity: Genetic Copy Number Variations Profiling Linked to Specific Metabolic Dysregulation Patterns
Source: Int J Mol Sci. 2025 May 16;26(10):4782. doi: 10.3390/ijms26104782 (PMC12112116; doi:10.3390/ijms26104782)
Supplement: Supplementary file 1 [file ijms-26-04782-s001.zip › figures/fig S1.pdf]

COFFALYSER

Sample report: 4

Sample type: Sample | Project: p220 | Experiment: 20220607 | Dye: 6-FAM | Performed by: Admin
Machine: ABI-3500 | Report date: 6/7/2022 | Run date: 6/7/2022 | Software Version: v.140721.1958 | Normal range: 0.7 - 1.3

Authorization	
Date	

**MLPA probe mix:**	P220-Obesity	**Nr of test probes:**	39/47	**FRSS:**	Bad 40%
**Lot number:**	B3-0919	**Nr of ref probes**	7/8	**FRMS:**	Warning 70%
**Sheet date:**	5/31/2022 9:45:02 AM	**DNA concentration:**	OK	**PSLP:**	OK 6%
**Control fragments:**	CF-003-[brown] QDX2 (A2-1	**DNA denaturation**	OK	**RSQ:**	OK
**Analysis method:**	Block SSC: On	**Expected gender:**	Female	**RPQ:**	Bad
**Used metric:**	Peak height	**Residual primer %**	OK 10%	**CAS:**	Warning 50%

Reference Samples: 1 | 2 | 3

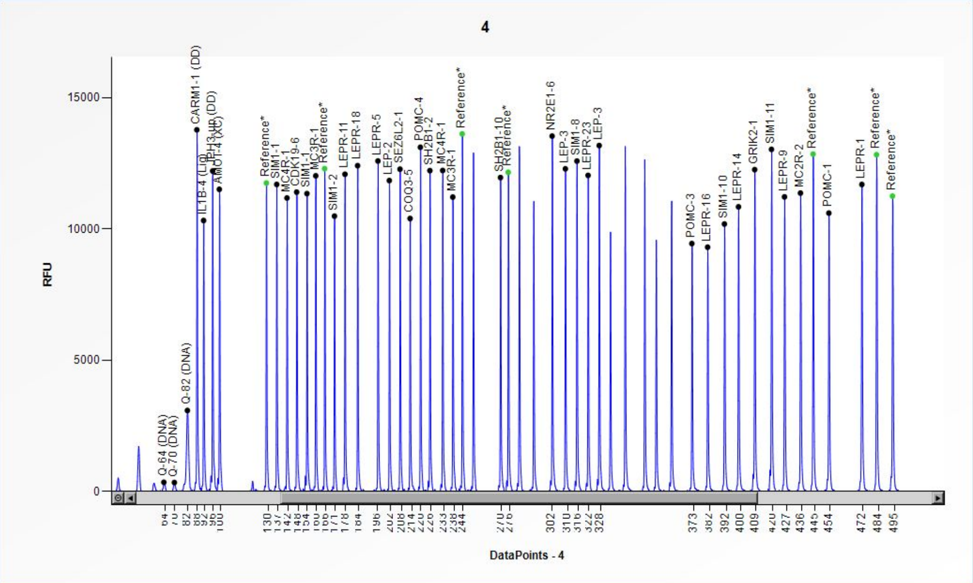

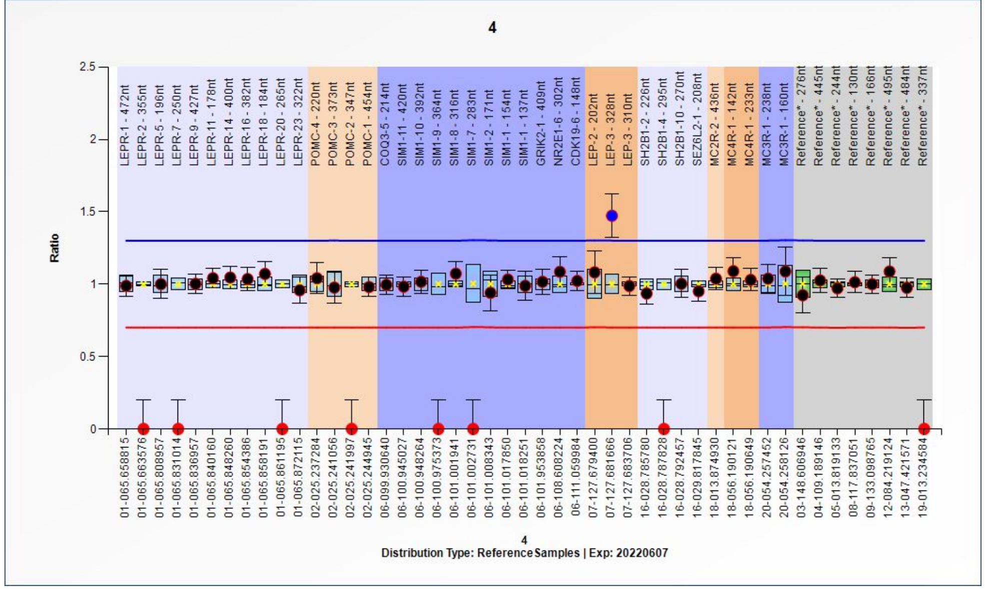

D [nt]	Gene-Exon	Chr.band	hg18 loc.	Height	Area	Ratio[H]	Stdev	[REF]	[Sam]	Width	d[nt]
472	LEPR-1	01p31.3	01-065.658815	11689	71250	0.99	0.04	=	=	57	-0.1
355	LEPR-2	01p31.3	01-065.663576	0	0	0	0	<<**	<<**	0	0.0
196	LEPR-5	01p31.3	01-065.808957	12583	56530	1	0.05	=	=	42	0.8
250	LEPR-7	01p31.3	01-065.831014	0	0	0	0	<<**	<<**	0	0.0
427	LEPR-9	01p31.3	01-065.836957	11217	62991	1	0.03	=	=	52	0.3
178	LEPR-11	01p31.3	01-065.840160	12079	57302	1.04	0.03	=	=	45	1.1
400	LEPR-14	01p31.3	01-065.848260	10838	60550	1.05	0.04	=	=	52	-0.2
382	LEPR-16	01p31.3	01-065.854386	9303	49546	1.04	0.04	=	=	40	-0.9
184	LEPR-18	01p31.3	01-065.858191	12406	55101	1.07	0.04	=	=	34	1.1
265	LEPR-20	01p31.3	01-065.861195	0	0	0	0	<<**	<<**	0	0.0
322	LEPR-23	01p31.3	01-065.872115	12036	59270	0.96	0.05	=	=	34	-0.2
220	POMC-4	02p23.3	02-025.237284	13106	59257	1.04	0.05	=	=	29	-0.1
373	POMC-3	02p23.3	02-025.241056	9440	51545	0.98	0.05	=	=	75	-1.7
347	POMC-2	02p23.3	02-025.241997	0	0	0	0	<<**	<<**	0	0.0
454	POMC-1	02p23.3	02-025.244945	10600	63019	0.98	0.03	=	=	61	0.2
214	COQ3-5	06q16.3	06-099.930640	10394	49529	1	0.03	=	=	43	0.2
420	SIM1-11	06q16.3	06-100.945027	13025	74717	0.98	0.03	=	=	35	0.2
392	SIM1-10	06q16.3	06-100.948264	10185	55475	1.02	0.04	=	=	66	-0.4
364	SIM1-9	06q16.3	06-100.975373	0	0	0	0	<<**	<<**	0	0.0
316	SIM1-8	06q16.3	06-101.001941	12581	63769	1.07	0.04	=	=	36	-0.5
283	SIM1-7	06q16.3	06-101.002731	0	0	0	0	<<**	<<**	0	0.0
171	SIM1-2	06q16.3	06-101.008343	10484	48283	0.94	0.06	=	=	40	1.2
154	SIM1-1	06q16.3	06-101.017850	11342	53381	1.03	0.03	=	=	41	1.0
137	SIM1-1	06q16.3	06-101.018251	11691	53326	0.99	0.05	=	=	39	0.4
409	GRIK2-1	06q16.3	06-101.953858	12254	70644	1.02	0.04	=	=	65	0.1
302	NR2E1-6	06q21	06-108.608224	13530	68902	1.09	0.05	=	=	62	0.8
148	CDK19-6	06q21	06-111.059984	11400	54186	1.02	0.03	=	=	39	0.8
202	LEP-2	07q32.1	07-127.679400	11839	53178	1.08	0.07	=	=	30	0.7
328	LEP-3	07q32.1	07-127.681666	13171	67785	1.47	0.08	>>*	>>*	53	0.3
310	LEP-3	07q32.1	07-127.683706	12285	60008	0.99	0.03	=	=	46	-0.4
226	SH2B1-2	16p11.2	16-028.785780	12217	56263	0.94	0.04	=	=	30	-0.4
295	SH2B1-4	16p11.2	16-028.787828	0	0	0	0	<<**	<<**	0	0.0
270	SH2B1-10	16p11.2	16-028.792457	11953	56205	1	0.05	=	=	41	0.5
208	SEZ6L2-1	16p11.2	16-029.817845	12272	55537	0.95	0.03	=	=	42	0.4
436	MC2R-2	18p11.21	18-013.874930	11362	65550	1.04	0.04	=	=	60	0.3
142	MC4R-1	18q21.32	18-056.190121	11175	50654	1.09	0.04	=	=	40	0.5
233	MC4R-1	18q21.32	18-056.190649	12222	58310	1.03	0.04	=	=	40	-1.0
238	MC3R-1	20q13.2	20-054.257452	11209	53952	1.04	0.05	=	=	38	-1.4
160	MC3R-1	20q13.2	20-054.258126	12018	54854	1.09	0.08	=	=	31	1.1
276	Reference*	03q24	03-148.606946	12152	60306	0.92	0.06	=	=	37	1.9
445	Reference*	04q25	04-109.189146	12845	73182	1.03	0.04	=	=	47	0.3
244	Reference*	05p15.2	05-013.819133	13614	63372	0.97	0.03	=	=	39	-1.8
130	Reference*	08q24.11	08-117.837051	11745	55875	1.01	0.04	=	=	49	0.3
166	Reference*	09q34.13	09-133.098765	12290	56235	1	0.03	=	=	35	1.3
495	Reference*	12q21.31	12-084.219124	11247	69328	1.09	0.05	=	=	62	0.0
484	Reference*	13q14.2	13-047.421571	12824	75479	0.98	0.03	=	=	58	0.0
337	Reference*	19p13.13	19-013.234584	0	0	0	0	<<**	<<**	0	0.0
**Median value all probe values:**				**12018**	**57302**	**1.02**	**0.04**			**41**	**0.27**

Normal range: 0.7 - 1.3 Essential information on the use of this product is present in the product description which is available on http://www.mlpa.com. For questions, mailto:info@mlpa.com.
The 148 nt probe 03945-L03403, 214 nt probe 16153-L19387, 302 nt probe 03939-L09973, 409 nt probe 16154-L18338 are a "flanking probe". It is included to facilitate the determination of the extent of a deletion/duplication. Copy number alterations of flanking and reference probes are unlikely to be related to the condition tested. *** The signal o f the 238 nt probe 08884-L08940 might be influenced by SNP rs61736060 (non-validated). In case of apparent deletions, it is recommended to sequence the region targeted by this probe.... Please see the product description for all notifications.
