# Supplementary material for: Towards Precision Medicine in Obesity: Genetic Copy Number Variations Profiling Linked to Specific Metabolic Dysregulation Patterns
Source: Int J Mol Sci. 2025 May 16;26(10):4782. doi: 10.3390/ijms26104782 (PMC12112116; doi:10.3390/ijms26104782)
Supplement: Supplementary file 1 [file ijms-26-04782-s001.zip › figures/fig S5.pdf]

COFFALYSER

Sample report: 53

Sample type: Sample | Project: 20220727 | Experiment: 20220727 tura 2 | Dye: 6-FAM | Performed by: Admin
Machine: ABI-3500 | Report date: 7/27/2022 | Run date: 7/27/2022 | Software Version: v.140721.1958 | Normal range: 0.7 - 1.3

Authorization	
Date	

**MLPA probe mix:**	P220-Obesity	**Nr of test probes:**	47/47	**FRSS:**	OK 100%
**Lot number:**	B3-0919	**Nr of ref probes**	8/8	**FRMS:**	OK 100%
**Sheet date:**	5/31/2022 9:45:02 AM	**DNA concentration:**	OK	**PSLP:**	OK 7%
**Control fragments:**	CF-003-[brown] QDX2 (A2-1	**DNA denaturation**	OK	**RSQ:**	OK
**Analysis method:**	Block SSC: On	**Expected gender:**	Female	**RPQ:**	OK
**Used metric:**	Peak height	**Residual primer %**	OK 13%	**CAS:**	OK 100%

Reference Samples: C | I | O

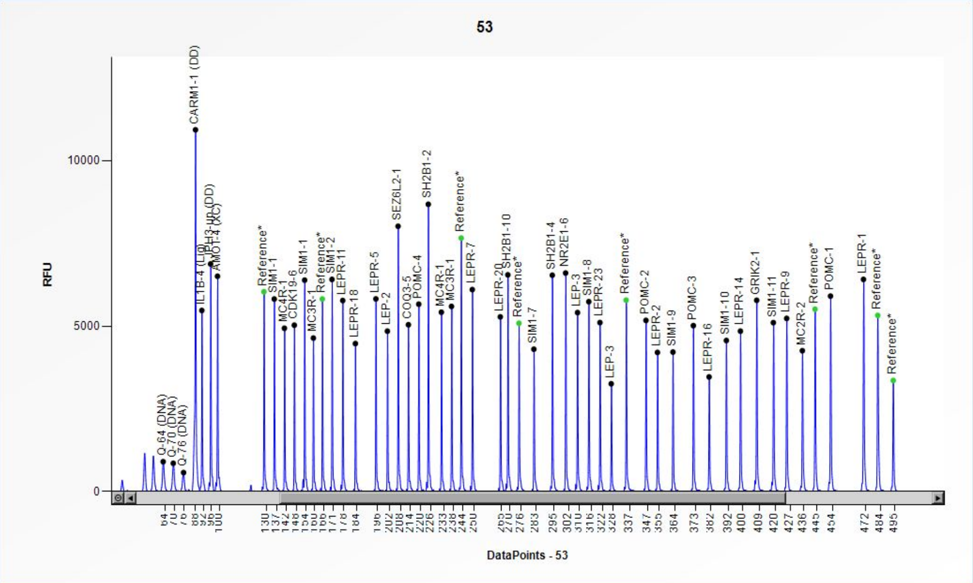

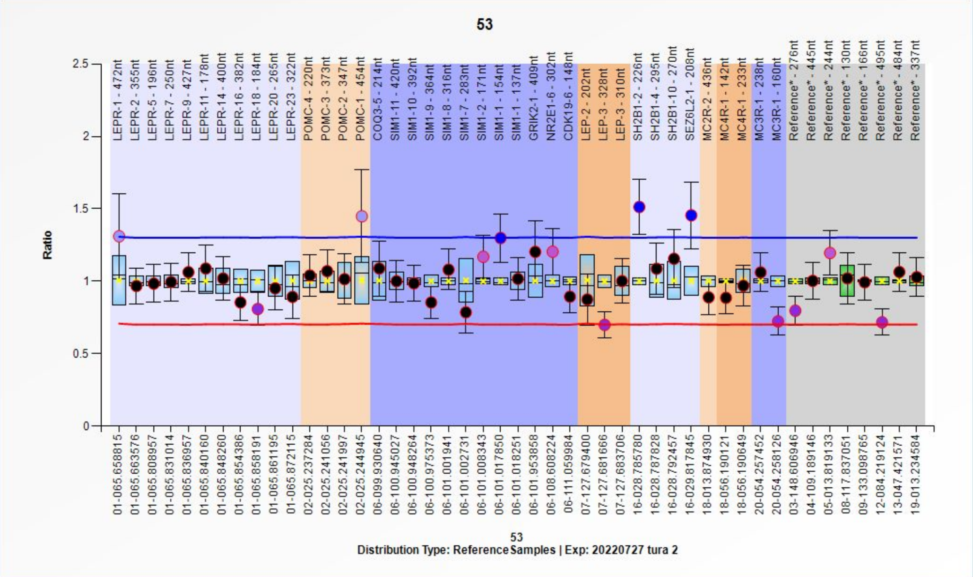

D [nt]	Gene-Exon	Chr.band	hg18 loc.	Height	Area	Ratio[H]	Stdev	[REF]	[Sam]	Width	d[nt]
472	LEPR-1	01p31.3	01-065.658815	6412	40705	**1.31**	**0.15**	**>***	?	60	0.1
355	LEPR-2	01p31.3	01-065.663576	4205	22071	*0.97*	0.06	=	=	46	0.1
196	LEPR-5	01p31.3	01-065.808957	5823	25747	*0.98*	0.06	=	=	42	0.0
250	LEPR-7	01p31.3	01-065.831014	6104	28324	*0.99*	0.06	=	=	43	0.0
427	LEPR-9	01p31.3	01-065.836957	5233	29990	*1.06*	0.07	=	=	64	0.0
178	LEPR-11	01p31.3	01-065.840160	5773	26043	*1.09*	0.08	=	=	40	0.0
400	LEPR-14	01p31.3	01-065.848260	4848	27205	*1.02*	0.08	=	=	62	0.0
382	LEPR-16	01p31.3	01-065.854386	3465	19254	*0.85*	0.06	=	=	44	0.1
184	LEPR-18	01p31.3	01-065.858191	4472	20789	*0.81*	0.06	**<<**	=	44	0.0
265	LEPR-20	01p31.3	01-065.861195	5280	25365	*0.95*	0.08	=	=	36	0.1
322	LEPR-23	01p31.3	01-065.872115	5107	25990	*0.89*	0.08	=	=	50	0.1
220	POMC-4	02p23.3	02-025.237284	5663	25939	*1.04*	0.07	=	=	46	-0.1
373	POMC-3	02p23.3	02-025.241056	5015	27047	*1.07*	0.07	=	=	64	0.0
347	POMC-2	02p23.3	02-025.241997	5173	26489	*1.01*	0.09	=	=	55	0.0
454	POMC-1	02p23.3	02-025.244945	5907	35110	**1.45**	**0.16**	**>***	?	45	0.0
214	COQ3-5	06q16.3	06-099.930640	5042	24044	*1.09*	0.1	=	=	46	-0.1
420	SIM1-11	06q16.3	06-100.945027	5105	30715	*1*	0.07	=	=	62	0.1
392	SIM1-10	06q16.3	06-100.948264	4566	25409	*0.99*	0.06	=	=	64	0.0
364	SIM1-9	06q16.3	06-100.975373	4216	22157	*0.85*	0.06	=	=	57	0.0
316	SIM1-8	06q16.3	06-101.001941	5738	28977	*1.08*	0.07	=	=	49	0.0
283	SIM1-7	06q16.3	06-101.002731	4303	22222	*0.79*	0.07	=	=	46	0.1
171	SIM1-2	06q16.3	06-101.008343	6411	28392	*1.17*	0.07	**>>**	=	41	0.1
154	SIM1-1	06q16.3	06-101.017850	6389	29914	***1.3***	0.08	**>>***	?	35	0.0
137	SIM1-1	06q16.3	06-101.018251	5819	26199	*1.02*	0.07	=	=	49	0.0
409	GRIK2-1	06q16.3	06-101.953858	5780	34432	*1.2*	**0.11**	=	=	71	0.0
302	NR2E1-6	06q21	06-108.608224	6604	33033	*1.2*	0.08	**>>**	=	57	0.0
148	CDK19-6	06q21	06-111.059984	5028	22061	*0.89*	0.06	=	=	40	0.0
202	LEP-2	07q32.1	07-127.679400	4847	21916	*0.87*	0.09	=	=	46	0.0
328	LEP-3	07q32.1	07-127.681666	3258	16544	*0.7*	0.05	**<<**	=	45	0.1
310	LEP-3	07q32.1	07-127.683706	5409	27886	*1*	0.08	=	=	43	-0.1
226	SH2B1-2	16p11.2	16-028.785780	8681	40141	***1.51***	0.1	**>>***	>*	52	0.0
295	SH2B1-4	16p11.2	16-028.787828	6538	32714	*1.09*	0.09	=	=	56	0.0
270	SH2B1-10	16p11.2	16-028.792457	6549	31547	*1.16*	0.1	=	=	39	0.0
208	SEZ6L2-1	16p11.2	16-029.817845	8017	36546	***1.46***	**0.12**	**>>***	?	49	0.0
436	MC2R-2	18p11.21	18-013.874930	4256	25690	*0.89*	0.06	=	=	59	0.0
142	MC4R-1	18q21.32	18-056.190121	4936	22866	*0.89*	0.06	=	=	42	0.1
233	MC4R-1	18q21.32	18-056.190649	5421	24691	*0.97*	0.07	=	=	51	0.0
238	MC3R-1	20q13.2	20-054.257452	5595	26440	*1.06*	0.07	=	=	42	0.0
160	MC3R-1	20q13.2	20-054.258126	4640	21294	*0.72*	0.05	**<<**	=	36	0.0
276	Reference*	03q24	03-148.606946	5087	25978	*0.8*	0.05	**<<**	=	57	0.0
445	Reference*	04q25	04-109.189146	5512	32120	*1*	0.06	=	=	41	0.0
244	Reference*	05p15.2	05-013.819133	7661	35428	*1.2*	0.08	**>>**	=	42	-0.1
130	Reference*	08q24.11	08-117.837051	6039	27884	*1.02*	0.09	=	=	51	0.0
166	Reference*	09q34.13	09-133.098765	5819	25984	*0.99*	0.06	=	=	36	0.0
495	Reference*	12q21.31	12-084.219124	3359	20646	*0.72*	0.05	**<<**	=	51	-0.1
484	Reference*	13q14.2	13-047.421571	5325	35742	*1.06*	0.07	=	=	55	-0.1
337	Reference*	19p13.13	19-013.234584	5785	30840	*1.03*	0.07	=	=	82	0.1
**Median value all probe values:**				**5409**	**26440**	**1.01**	**0.07**			**46**	**0.02**

Normal range: 0.7 - 1.3 Essential information on the use of this product is present in the product description which is available on http://www.mlpa.com. For questions, mailto:info@mlpa.com.
The 148 nt probe 03945-L03403, 214 nt probe 16153-L19387, 302 nt probe 03939-L09973, 409 nt probe 16154-L18338 are a "flanking probe". It is included to facilitate the determination of the extent of a deletion/duplication. Copy number alterations of flanking and reference probes are unlikely to be related to the condition tested. *** The signal o f the 238 nt probe 08884-L08940 might be influenced by SNP rs61736060 (non-validated). In case of apparent deletions, it is recommended to sequence the region targeted by this probe.... Please see the product description for all notifications.
