# Supplementary material for: Towards Precision Medicine in Obesity: Genetic Copy Number Variations Profiling Linked to Specific Metabolic Dysregulation Patterns
Source: Int J Mol Sci. 2025 May 16;26(10):4782. doi: 10.3390/ijms26104782 (PMC12112116; doi:10.3390/ijms26104782)
Supplement: Supplementary file 1 [file ijms-26-04782-s001.zip › figures/fig S4.pdf]

Sample report: 47

Sample type: Sample | Project: 20220727 | Experiment: 20220727 tura 2 | Dye: 6-FAM | Performed by: Admin
Machine: ABI-3500 | Report date: 7/27/2022 | Run date: 7/27/2022 | Software Version: v.140721.1958 | Normal range: 0.7 - 1.3

Authorization	
Date	

**MLPA probe mix:**	P220-Obesity	**Nr of test probes:**	47/47	**FRSS:**	OK? 85%
**Lot number:**	B3-0919	**Nr of ref probes**	8/8	**FRMS:**	OK 100%
**Sheet date:**	5/31/2022 9:45:02 AM	**DNA concentration:**	OK	**PSLP:**	Warning -19%
**Control fragments:**	CF-003-[brown] QDX2 (A2-1	**DNA denaturation**	OK	**RSQ:**	OK
**Analysis method:**	Block SSC: On	**Expected gender:**	Male	**RPQ:**	Warning
**Used metric:**	Peak height	**Residual primer %**	OK 13%	**CAS:**	OK? 85%

Reference Samples: C | I | O

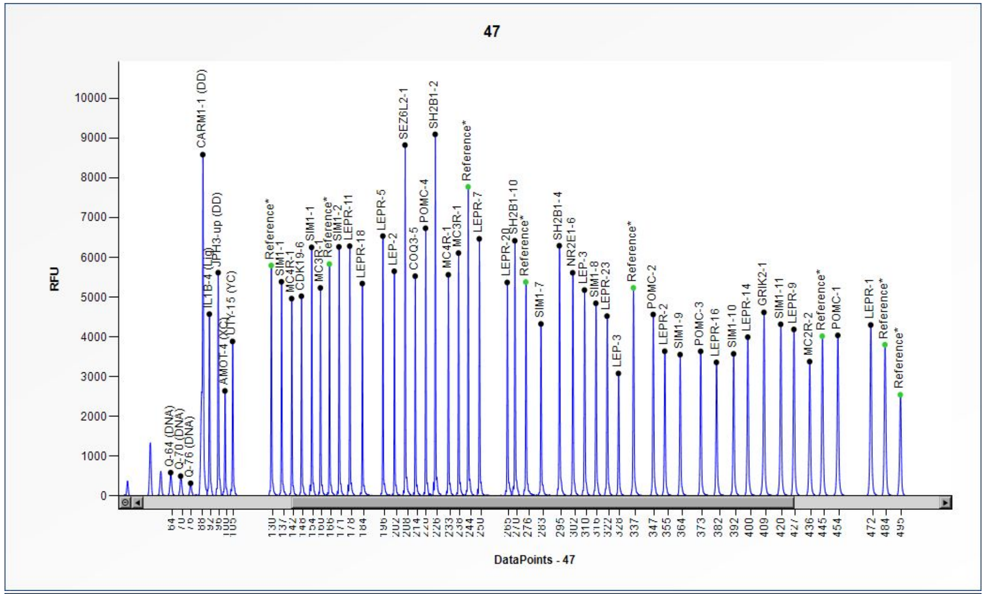

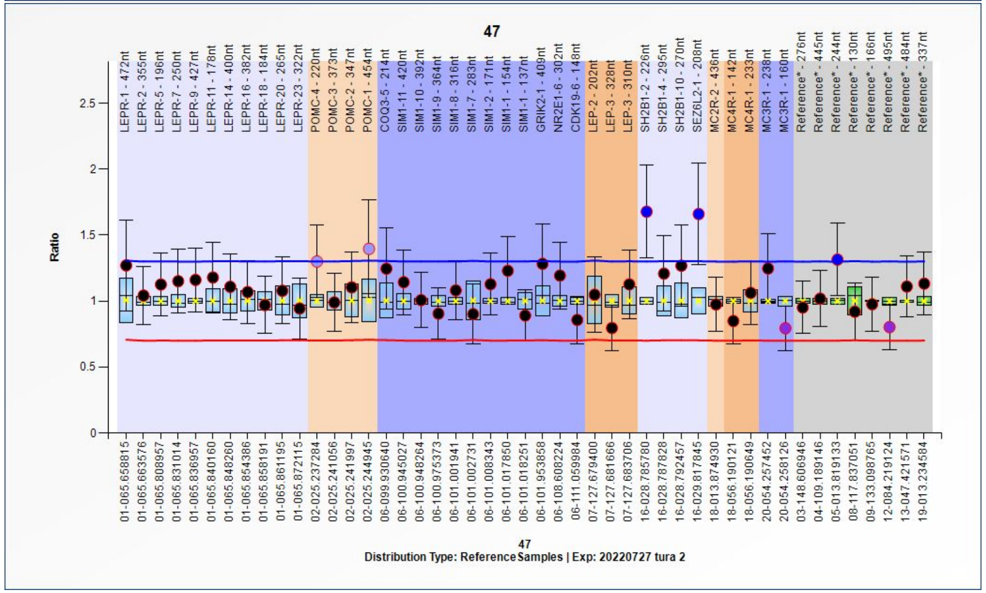

D [nt]	Gene-Exon	Chr.band	hg18 loc.	Height	Area	Ratio[H]	Stdev	[REF]	[Sam]	Width	d[nt]
472	LEPR-1	01p31.3	01-065.658815	4303	42713	*1.27*	**0.17**	=	=	65	0.1
355	LEPR-2	01p31.3	01-065.663576	3642	26886	*1.04*	**0.11**	=	=	68	0.0
196	LEPR-5	01p31.3	01-065.808957	6537	29696	*1.13*	**0.12**	=	=	41	-0.1
250	LEPR-7	01p31.3	01-065.831014	6467	32148	*1.15*	**0.12**	=	=	45	0.0
427	LEPR-9	01p31.3	01-065.836957	4190	37147	*1.16*	**0.12**	=	=	46	0.0
178	LEPR-11	01p31.3	01-065.840160	6284	29665	*1.18*	**0.13**	=	=	48	-0.1
400	LEPR-14	01p31.3	01-065.848260	3997	33501	*1.11*	**0.13**	=	=	65	0.0
382	LEPR-16	01p31.3	01-065.854386	3362	27162	*1.07*	**0.12**	=	=	59	0.0
184	LEPR-18	01p31.3	01-065.858191	5343	23865	*0.97*	**0.11**	=	=	39	0.0
265	LEPR-20	01p31.3	01-065.861195	5371	29162	*1.08*	**0.13**	=	=	38	0.1
322	LEPR-23	01p31.3	01-065.872115	4525	30007	*0.94*	**0.12**	=	=	49	0.1
220	POMC-4	02p23.3	02-025.237284	6732	29753	**1.3**	**0.14**	>*	?	28	0.0
373	POMC-3	02p23.3	02-025.241056	3639	28461	*0.99*	**0.11**	=	=	60	0.0
347	POMC-2	02p23.3	02-025.241997	4565	32311	*1.1*	**0.13**	=	=	64	0.0
454	POMC-1	02p23.3	02-025.244945	4040	39008	**1.4**	**0.19**	>*	?	81	0.0
214	COQ3-5	06q16.3	06-099.930640	5529	24747	*1.25*	**0.15**	=	=	44	0.0
420	SIM1-11	06q16.3	06-100.945027	4319	39187	*1.14*	**0.12**	=	=	77	0.0
392	SIM1-10	06q16.3	06-100.948264	3577	29781	*1.01*	**0.11**	=	=	71	0.0
364	SIM1-9	06q16.3	06-100.975373	3556	26574	*0.91*	0.1	=	=	59	0.0
316	SIM1-8	06q16.3	06-101.001941	4847	30884	*1.08*	**0.11**	=	=	40	0.0
283	SIM1-7	06q16.3	06-101.002731	4329	25878	*0.9*	**0.11**	=	=	48	0.0
171	SIM1-2	06q16.3	06-101.008343	6266	29418	*1.13*	**0.12**	=	=	46	0.1
154	SIM1-1	06q16.3	06-101.017850	6254	30112	*1.23*	**0.13**	=	=	41	0.1
137	SIM1-1	06q16.3	06-101.018251	5388	26800	*0.89*	0.1	=	=	48	0.1
409	GRIK2-1	06q16.3	06-101.953858	4619	40994	*1.28*	**0.15**	=	=	72	0.0
302	NR2E1-6	06q21	06-108.608224	5614	34061	*1.19*	**0.13**	=	=	47	0.0
148	CDK19-6	06q21	06-111.059984	5025	23817	*0.86*	0.09	=	=	40	0.1
202	LEP-2	07q32.1	07-127.679400	5652	24912	*1.05*	**0.14**	=	=	46	0.0
328	LEP-3	07q32.1	07-127.681666	3084	20440	*0.8*	0.08	=	=	42	0.0
310	LEP-3	07q32.1	07-127.683706	5179	32088	*1.13*	**0.13**	=	=	52	0.0
226	SH2B1-2	16p11.2	16-028.785780	9094	42334	***1.68***	**0.18**	>>*	>*	50	0.0
295	SH2B1-4	16p11.2	16-028.787828	6294	38113	*1.21*	**0.14**	=	=	56	0.0
270	SH2B1-10	16p11.2	16-028.792457	6420	35577	*1.27*	**0.16**	=	=	48	0.0
208	SEZ6L2-1	16p11.2	16-029.817845	8824	39676	***1.66***	**0.19**	>>*	>*	50	0.0
436	MC2R-2	18p11.21	18-013.874930	3382	31223	*0.97*	0.1	=	=	59	0.0
142	MC4R-1	18q21.32	18-056.190121	4965	23764	*0.85*	0.09	=	=	44	0.1
233	MC4R-1	18q21.32	18-056.190649	5564	27532	*1.06*	**0.12**	=	=	56	0.0
238	MC3R-1	20q13.2	20-054.257452	6110	29216	*1.25*	**0.13**	=	=	40	0.0
160	MC3R-1	20q13.2	20-054.258126	5234	24496	*0.79*	0.08	<<	=	36	0.0
276	Reference*	03q24	03-148.606946	5381	32115	*0.95*	0.1	=	=	64	0.1
445	Reference*	04q25	04-109.189146	4021	37375	*1.02*	**0.11**	=	=	56	0.0
244	Reference*	05p15.2	05-013.819133	7774	38227	***1.31***	**0.14**	>>*	>*	53	0.0
130	Reference*	08q24.11	08-117.837051	5797	30759	*0.92*	**0.11**	=	=	51	0.0
166	Reference*	09q34.13	09-133.098765	5833	26640	*0.98*	0.1	=	=	43	0.0
495	Reference*	12q21.31	12-084.219124	2548	25325	*0.8*	0.08	<<	=	48	0.0
484	Reference*	13q14.2	13-047.421571	3805	38886	*1.11*	**0.12**	=	=	62	0.0
337	Reference*	19p13.13	19-013.234584	5235	36665	*1.13*	**0.12**	=	=	66	0.0
**Median value all probe values:**				**5234**	**30007**	**1.1**	**0.12***			**49**	**0**

Normal range: 0.7 - 1.3 Essential information on the use of this product is present in the product description which is available on http://www.mlpa.com. For questions, mailto:info@mlpa.com.
The 148 nt probe 03945-L03403, 214 nt probe 16153-L19387, 302 nt probe 03939-L09973, 409 nt probe 16154-L18338 are a "flanking probe". It is included to facilitate the determination of the extent of a deletion/duplication. Copy number alterations of flanking and reference probes are unlikely to be related to the condition tested. *** The signal o f the 238 nt probe 08884-L08940 might be influenced by SNP rs6173606 0 (non-validated). In case of apparent deletions, it is recommended to sequence the region targeted by this probe.... Please see the product description for all notifications.
